# Alagille-like syndrome with surprising karyotype: a case report

**DOI:** 10.1186/s13256-023-03810-7

**Published:** 2023-04-26

**Authors:** S. Amimoto, M. Ishii, K. Tanaka, S. Araki, M. Kuwamura, S. Suga, E. Kondo, E. Shibata, K. Kusuhara, K. Yoshino

**Affiliations:** 1 Department of Obstetrics and Gynecology, University of Occupational and Environmental Health, Kitakyushu, Japan; 2 Department of Pediatrics, Kitakyushu General Hospital, 1-1 Higashijonochou, Kokurakita-Ku, Kitakyushu-City, 802-8517 Japan; 3 Department of Pediatrics, University of Occupational and Environmental Health, Kitakyushu, Japan

**Keywords:** Alagille syndrome, 5p-syndrome, 6p trisomy, Hepatic disorder, Case report

## Abstract

**Background:**

Chromosome 5p partial monosomy (5p-syndrome) and chromosome 6p partial trisomy are chromosomal abnormalities that result in a variety of symptoms, but liver dysfunction is not normally one of them. Alagille syndrome (OMIM #118450) is a multisystem disorder that is defined clinically by hepatic bile duct paucity and cholestasis, in association with cardiac, skeletal, and ophthalmologic manifestations, and characteristic facial features. Alagille syndrome is caused by mutations in *JAG1* on chromosome 20 or *NOTCH2* on chromosome 1. Here, we report a preterm infant with karyotype 46,XX,der(5)t(5,6)(p15.2;p22.3) and hepatic dysfunction, who was diagnosed as having incomplete Alagille syndrome.

**Case presentation:**

The Japanese infant was diagnosed based on the cardiac abnormalities, ocular abnormalities, characteristic facial features, and liver pathological findings. Analysis of the *JAG1* and *NOTCH* sequences failed to detect any mutations in these genes.

**Conclusions:**

These results suggest that, besides the genes that are known to be responsible for Alagille syndrome, other genetic mutations also may cause Alagille syndrome.

## Background

The 5p-syndrome is a chromosomal abnormality syndrome that is associated with partial deletion of the short arm of chromosome 5. The prevalence of the 5p-syndrome is 1 in 50,000 live births. Low birth weight (< 2500 g), growth retardation, and a high-pitched cat-like cry during the neonatal period and infancy have been observed in children with the 5p-syndrome. Hypotonia, moderate to severe mental retardation, and characteristic facial features such as microcephaly, round face, interocular separation, and low-set ears have also been observed [1].

Partial trisomy of the short arm of chromosome 6 is a rare chromosomal abnormality, with < 50 cases reported worldwide [2]. Characteristic phenotypes include low birth weight, developmental delay or growth retardation, mild to severe craniofacial abnormalities, eating disorder, recurrent respiratory infections, congenital heart disease, and renal abnormalities [3].

Alagille syndrome (AGS) is characterized by five main clinical abnormalities: liver dysfunction (cholestatic jaundice caused by paucity of bile ducts), cardiac abnormalities, spine anomalies, eye abnormalities, and characteristic facial features. Patients with these five clinical features are diagnosed with complete AGS, and patients with three or four of the features are diagnosed with incomplete AGS [4].The prevalence of AGS is 1 in 70,000 live births [5].

Hepatic disorders such as AGS with 5p monosomy or 6p trisomy have not been reported so far. The main clinical features of 5p monosomy, 6p trisomy, and Alagille syndrome are presented in Table 1. Here, we report an infant with 5p monosomy and 6p trisomy, complicated by refractory liver failure.

**Table 1:** Clinical features of 5p monosomy, 6p trisomy, and Alagille syndrome

	Current case	5p monosomy [1]	6p trisomy [3]	Alagille syndrome [4]
Hepatic disorders	Cholestatic liver (hypoplasia of the interlobular bile ducts, changes similar to giant cell tumor of hepatocytes and anomaly of blood vessels)	Unknown	Unknown	Cholestatic liver (paucity of interlobular bile duct, change similar to giant cell tumor of hepatocytes, anomaly of blood vessel)
Dysmorphic	Hawk/nosed, micrognathia, interocular dissociation, corneas with severe edema	Microretrognathia, epicanthal folds, broad nasal bridge, round face, interocular dissociation	Facial asymmetry, craniosynostosis, low/set ears, short nose, prominent nasal bridge, thin lips, small mouth, blepharophimosis, microphthalmia, and so on.	Prominent forehead, deep-set eyes with hypertelorism, pointed chin, saddle or straight nose, eye anomalies (posterior embryotoxon)
Development (physical and mental)	FGR, growth retardation	Mental retardation	FGR, growth retardation, mental retardation	FGR, failure to thrive
Congenital heart disease	PDA	VSD, PDA, ASD	PDA, ASD, VSD, PPS	PPS, TOF, CoA
Others	Cat-like cry	Cat-like cry, ACC, renal abnormalities	CAKUT, Blain malformation, seizures	Spine anomalies (butterfly vertebrae), renal abnormalities

*FGR* fetal growth restriction, *PDA* patent ductus artery, *VSD* ventricular septal defect, *ASD* atrial septal defect, *PPS* peripheral pulmonic stenosis, *TOF* tetralogy of Fallot, *CoA* coarctation of the abdominal aorta, *ACC* agenesis of corpus callosum

## Case presentation

The patient is a Japanese female infant who is the second child of a healthy non-consanguineous 34-year-old mother and 35-year-old father. The infant has a healthy sister, and the family history is unremarkable. The mother was admitted to the Center of Maternal, Fetal, and Neonatal Medicine, Hospital of the University of Occupational and Environmental Health, Kitakyushu, Japan at the end of the 31st week of gestation, because the pregnancy was complicated with severe symmetrical fetal growth restriction, decreased fetal movement, and dilatation of patent ductus venosus. The preterm infant was born by cesarean section at the 32nd week of gestation because of severe decrease in fetal movement. The infant had respiratory distress with Apgar scores of 5 and 8 at 1 and 5 minutes after birth, respectively, and required neonatal cardiopulmonary resuscitation. Thus, the patient was intubated after birth and was transferred to the Neonatal Intensive Care Unit. At birth, the infant’s weight, length, and head circumference measurements were 995 g [± 3.4 standard deviation (SD)], 34 cm (± 3.3 SD), 26.7 cm (± 1.6 SD).

To investigate the cause of the fetal growth restriction, we checked the infant for congenital TORCH infections (*Toxoplasma gondii*, rubella virus, cytomegalovirus, herpes simplex virus, varicella-zoster virus, and *Treponema*), but none were found.

**Fig. 1:**
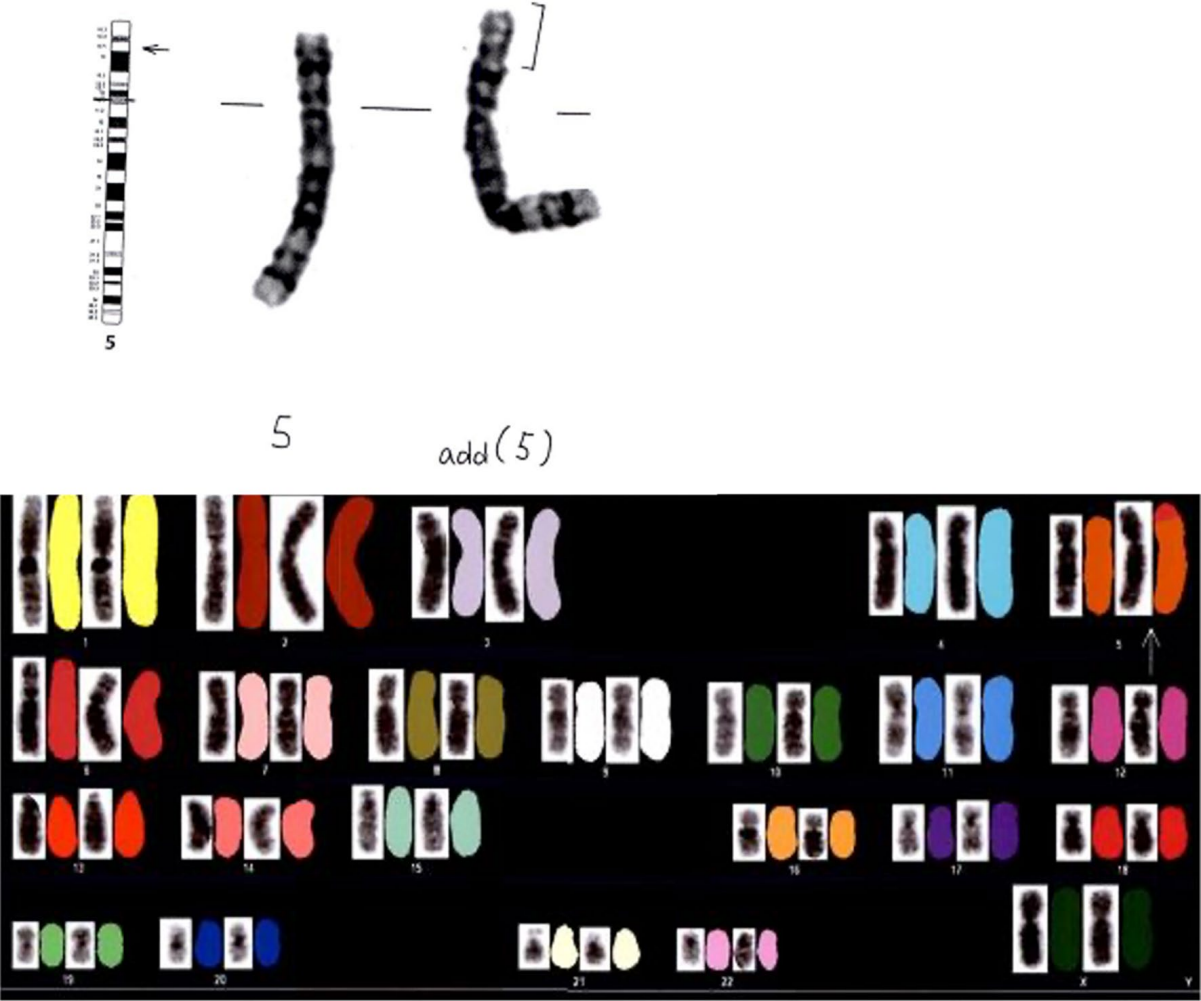
Chromosome G-banding and cytogenetic fluorescent in situ hybridization (FISH) show a deletion on chromosome 5p15.1-pter and additional 6p22.3 fragments on chromosome 5p and karyotype of 46,XX,der(5)t(5;6)(p15.2;p22.3), respectively

The patient’s face was characteristic (hawknosed, micrognathia, interocular dissociation) and the cry was cat-like. The chromosome G-banding analysis and cytogenetic fluorescent *in situ* hybridization analysis showed that the infant had a karyotype of 46,XX,der(5)t(5;6)(p15.2;p22.3) (Fig. 1). The patient’s parents agreed to undergo chromosome analysis after genetic counseling. The chromosome analysis showed that the mother’s chromosome was normal, whereas the father’s chromosome showed reciprocal translocation as 46,XY,t(5;6)(p15.2;p22.3).

The patient had patent ductus arteriosus (PDA) and left mild ventriculomegaly was detected by ultrasound. Tube feeding was started at day 1 after birth. The patient was treated with indomethacin at 2, 3, 4, 7, 8, and 9 days old for hemodynamically significant PDA. The PDA closed at 21 days old and no side effects of indomethacin were detected. However, the levels of aspartate aminotransferase, gamma-glutamyl transpeptidase, and direct bilirubin gradually increased from birth. The infant excreted a white-ish stool at 15 days old. Mutations in *JAG1* on chromosome 20 or *NOTCH2* on chromosome 1 are known to cause AGS. Therefore, we analyzed the *JAG1* and *NOTCH* sequences to detect mutations that can cause liver dysfunction, but no such mutations were found. We analyzed the bile acids and no abnormal bile acids were found. We performed magnetic resonance cholangiopancreatography (MRCP) to elucidate the etiology of cholangiolitic hepatitis and found that the structures of the patient’s liver, gallbladder, and bile duct were normal, but a congenital portosystemic shunt was observed. The patient was treated with ursodeoxycholic acid and phenobarbital, but her condition got worse after treatment. Furthermore, the PDA reopened when the patient was 25 days old.

Because the cause of the cholangiolitic hepatitis was unclear, we performed a liver biopsy. The pathological findings showed the patient had cholestatic liver disease with abnormal portal triad, compatible with AGS. We suspected that the patient had AGS-like or incomplete AGS. The patient’s hepatic condition worsened, the aspartate aminotransferase, gamma-glutamyl transpeptidase, and direct bilirubin levels were very high (peak values were 500 U/l, 326 U/l, and 19.0 mg/dl, respectively), and a blood coagulation disorder was discovered. The only treatment was liver transplantation, and therefore the patient was transferred for transplantation. PDA banding was required for the transplant, but the operation caused pulmonary bleeding and the infant died at 190 days old.

## Discussion and conclusions

We found that unbalanced reciprocal translocation between 5 and 6p (5p monosomy and 6p trisomy) may cause cholangiolitic hepatitis and AGS-like.

The 5p-syndrome is a chromosomal abnormality caused by deletion of the short arm of chromosome 5. The size of the deletion varies from the whole short arm to 5p15.3 (5–40 Mb) [6–9]. Clinical features include a high-pitched cat-like cry, microcephaly, facial features, and severe psychomotor and mental retardation. Approximately 80% of 5p-syndromes are *de novo* deletions, and approximately 10–15% are due to a parental translocation. The father of the patient in this study has reciprocal translocation (t(5;6)(p15.2;p22.3)).

Partial 6p trisomy is a rare chromosomal abnormality with variable phenotypes, including low birth weight with developmental delay or growth retardation, mild to severe anomaly of the face, eating difficulties, recurrent respiratory infections, congenital heart defects, and renal abnormalities [10].

The 5p monosomy and 6p trisomy have never been reported to have conjugated bilirubinemia, elevation of liver enzymes, or defects in biliary excretion (Table 1). The 5p monosomy that has been reported in many clinical reports may have a very low frequency of hepatic disorders. The 6p trisomy is a very rare chromosomal abnormality that also has not been associated with hepatic disorders. We suspect that the hepatic disorder such as AGS may be caused by unbalanced reciprocal translocation between 5 and 6p. Generally, unbalanced chromosomal rearrangements impact gene expression through a variety of different mechanisms. Chromosome rearrangements, both near to and far from breakpoints, can affect gene expression. Therefore, relocations can disrupt normal interactions not only at the derivative chromosomes, but also at other chromosomes, and affect gene expression [11].

AGS is an autosomal dominant disorder associated with abnormalities of the liver, heart, eye, and skeleton and a characteristic facial appearance. Syndromic bile duct paucity and arteriohepatic dysplasia, which are also observed in AGS, are significant causes of neonatal jaundice and cholestasis in older children.

AGS is characterized by reduction in the number of bile ducts on biopsy, leading to the obstruction of biliary flow (cholestasis) [4].

**Fig. 2:**
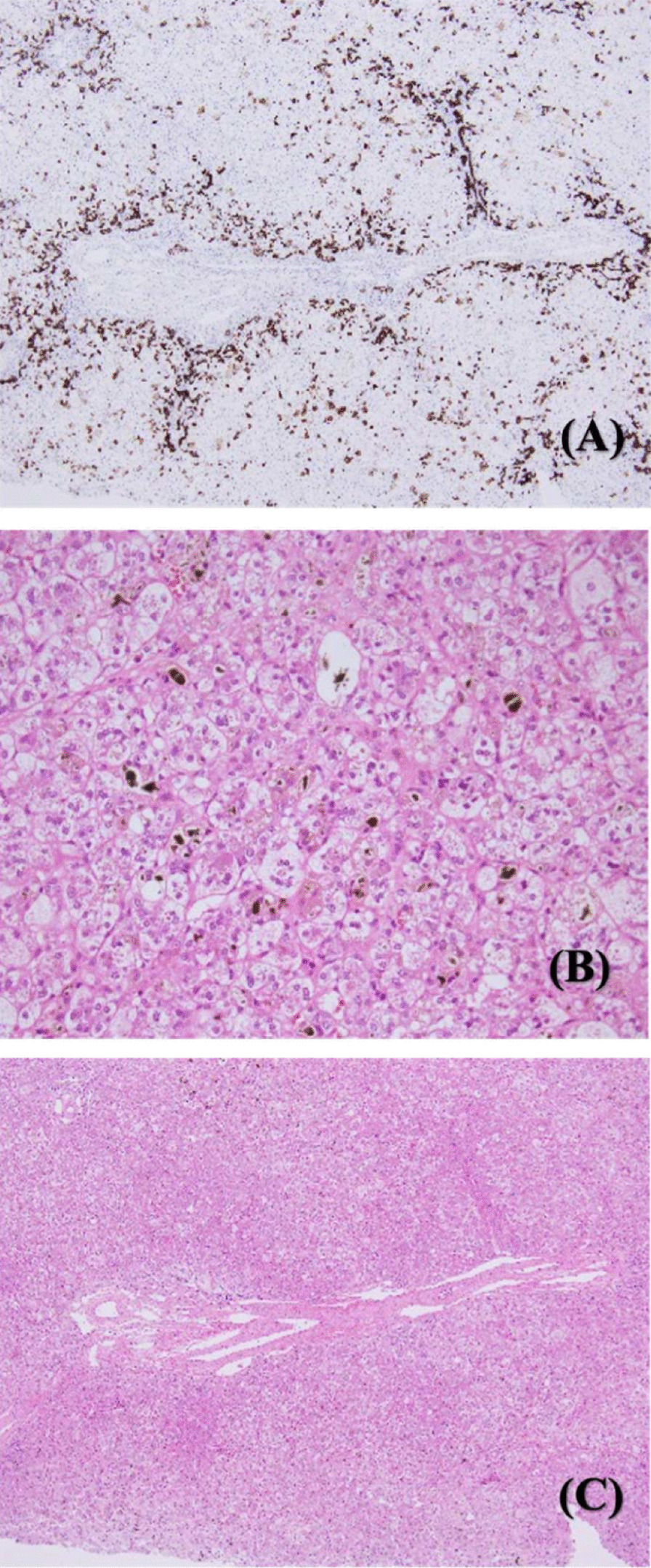
Liver biopsy images showing cholestatic liver with abnormal portal triad. **A** Paucity of interlobular bile duct. **B** Changes similar to giant cell tumor of hepatocytes. **C** Anomaly of blood vessels

In our patient, the gamma-glutamyl transpeptidase and direct bilirubin levels were elevated from birth, but the MRCP showed that the structures of the gallbladder and common bile duct were normal, and no biliary atresia was found. The bile acid analysis found no inborn errors of bile acid metabolism. The comprehensive genetic analysis for bile stasis disease did not identify any known genetic abnormalities, and the open liver biopsy confirmed the diagnosis. The pathological findings showed hypoplasia of the interlobular bile ducts (Fig. 2A), changes similar to giant cell tumor of hepatocytes (Fig. 2B) and anomaly of blood vessels (Fig. 2C).

Congenital heart disease has been reported in up to 90% of patients with AGS. The most common cardiac defect is peripheral pulmonic stenosis. Other reported cardiac defects include tetralogy of Fallot, ventricular septal defect, atrial septal defect, aortic stenosis, and coarctation of the aorta [4, 12, 13]. The most common skeletal abnormality is butterfly vertebrae, and ophthalmological findings include posterior embryotoxon [12, 13]. The range of facial features seen in patients with AGS include a prominent forehead, deep-set eyes with moderate hypertelorism, pointed chin, and saddle or straight nose with bulbous tip [4, 12, 13]. Other symptoms of AGS include renal anomalies, mental retardation, and delayed physical growth [4, 12]. No vertebral abnormalities were detected in our patient, but other cardiac abnormalities, ocular abnormalities, and characteristic facial features were observed. Because our patient had a histologically proven decrease in intrahepatic bile ducts, with at least three of the five main clinical abnormalities that characterize AGS, she was diagnosed with incomplete AGS [4, 12, 13]. In this patient, the phenotype was not representative, and therefore the liver biopsy was useful in confirming the diagnosis of AGS, even though liver biopsy is an invasive procedure.

AGS is caused by mutations in *JAG1*or *NOTCH2*; *JAG1*mutations have been identified in 94–96% and *NOTCH2* mutations have been identified in 1–2% of patients with AGS [14, 15]. The analysis of our patient’s DNA did not detect mutations in *JAG1* or *NOTCH*. To the best of our knowledge, four clinical reports of infants with AGS, who were found to have Williams syndrome, have been published. These infants were clinically diagnosed with AGS, but microarray analysis detected deletions at 7q11.23 [16]. We did not perform a whole genome analysis, but we speculate that incomplete AGS may be caused by a deletion at 5p15.2 and/or an insertion at 6p22.3. Our findings suggest that, besides the genes known to be responsible for AGS, one or more other genes can also cause the clinical features of AGS. To confirm this speculation, it is necessary to accumulate data of other patients with AGS in the future.

To conclude, we found that a patient with 5p monosomy and 6p trisomy had an abnormal liver structure, similar to AGS. The patient’s phenotype was uncharacteristic, and liver biopsy was found to be useful for AGS diagnosis, even in neonates. No mutations were detected in *JAG1*and *NOTCH2* but the patient had at least three of the five main clinical abnormalities that characterize AGS, thus she was diagnosed with incomplete AGS. Besides the genes known to be responsible for AGS, other genetic mutations also may lead to AGS.

## Data Availability

Not applicable.

## Abbreviation

AGS: Alagille syndrome
